# Supplementary material for: Neural representation of cytokines by vagal sensory neurons
Source: Nat Commun. 2025 Apr 24;16:3840. doi: 10.1038/s41467-025-59248-6 (PMC12019601; doi:10.1038/s41467-025-59248-6)
Supplement: Supplementary file 1 — Supplementary Information [file 41467_2025_59248_MOESM1_ESM.pdf]

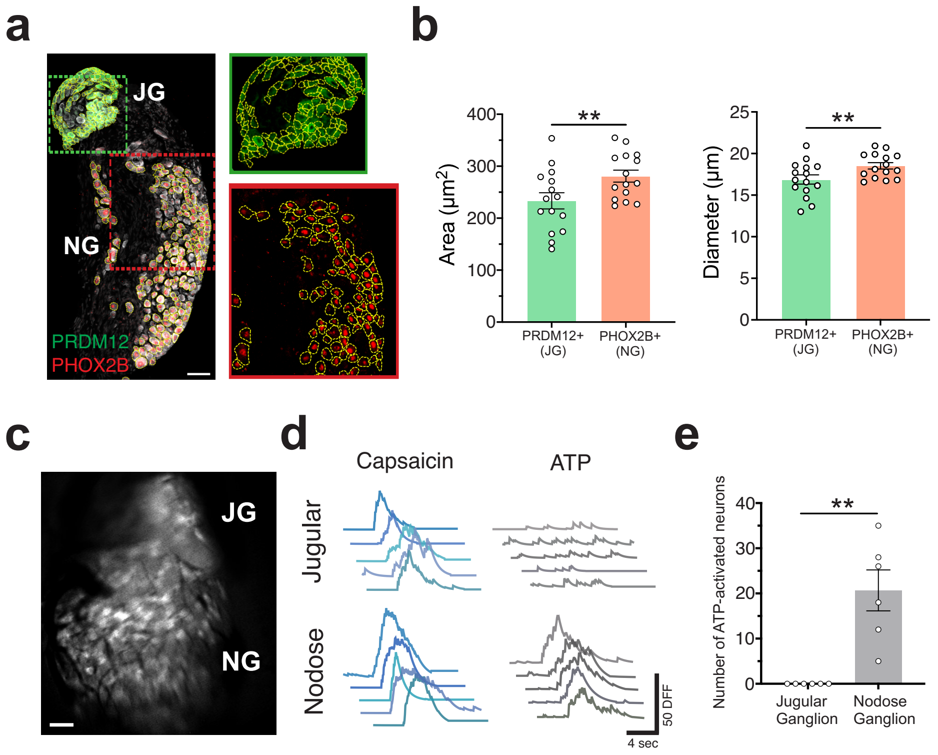

Supplementary Figure 1. Functional and structural examination of jugular and nodose ganglia neurons. a, Immunohistological assessment of distinct subsets between the jugular ganglia (JG) and nodose ganglia (NG) was performed to compare soma size. JG neurons are labeled by PRDM12 (green) and NG neurons are labeled by PHOX2B (red). Scale bar, 50 μm. **b**, Quantification and measurements using Zen Blue show that jugular ganglia cell bodies are significantly smaller than those in the nodose ganglia (per section, n =15). **c**, Representative Miniscope raw field-of-view image showing the jugular-nodose ganglia complex of a *Vglut2-GCaMP6f* mouse. Scale bar, 50 μm. **d**, Example traces of selective responses to vagus nerve administration of the TRPV1 channel activator capsaicin and the P2X receptor agonist α, β, methylene-ATP (α, β, m-ATP) in the jugular and nodose ganglia. Capsaicin elicited responses from sensory neurons in both the jugular and the nodose ganglia, whereas α, β, m-ATP selectively activated sensory neurons in only the nodose ganglia. **e**, Quantification of neuronal responses to α, β, m-ATP reveals that only nodose ganglia neurons are responsive (count per mouse, n = 6).

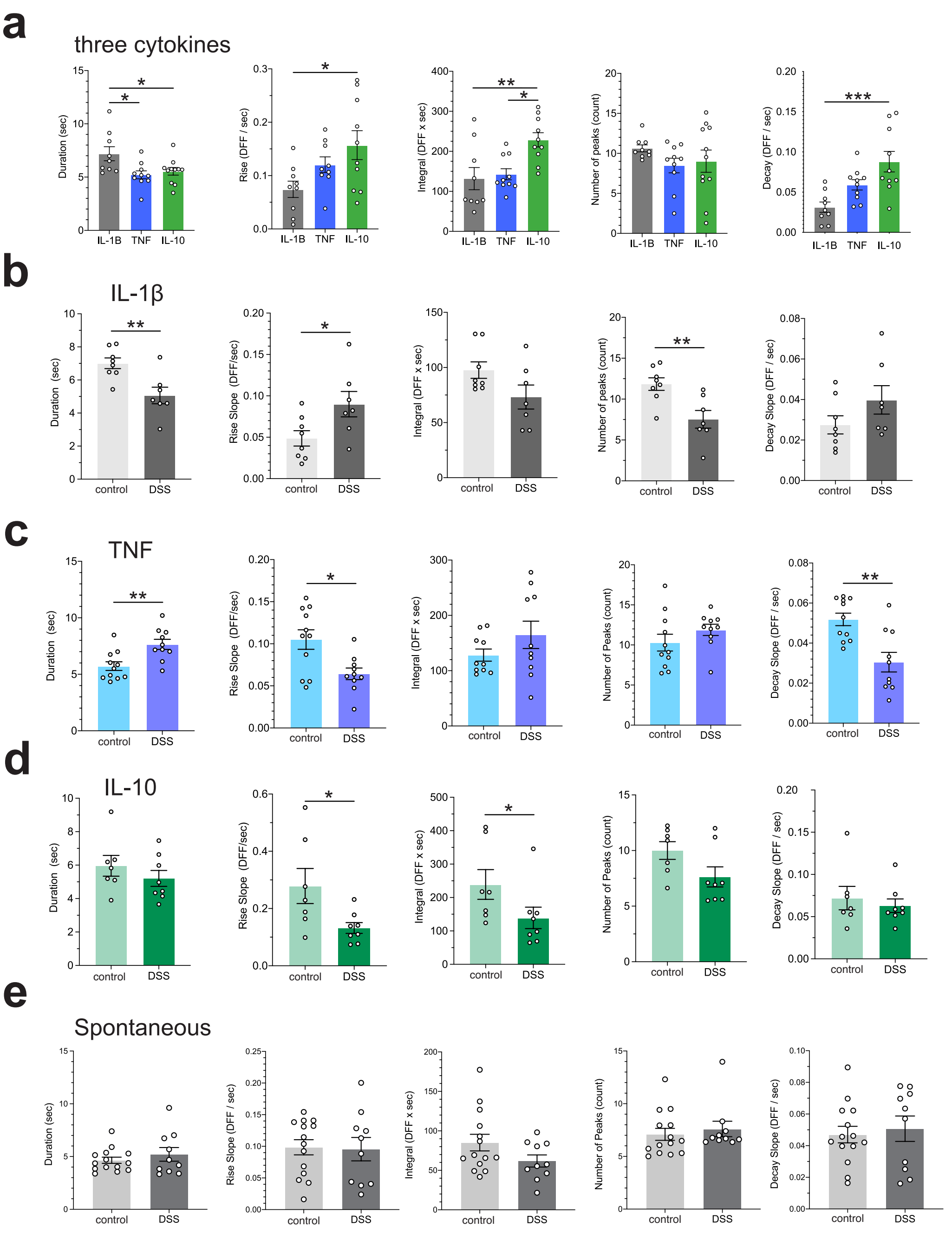

Supplementary Figure 2. Calcium transient response features. a, Comparison of additional features extracted from cytokine-evoked calcium transients (duration, rise, integral, number of peaks, decay; per mouse, mean ± SEM. n = 9, 10, 11, left to right) reveals cytokine-specific differences between aspects of response. **b**, DSS-induced colitis alters specific features of IL-1B-evoked responses with a reduction in duration and number of peaks and an increase rise slope (per mouse, n = 8, 7). **c**, DSS group responses to TNF application show alterations in several features including increased duration, and reduced rise and decay slope (per mouse, n = 11,10). **d**, IL-10 responses in the DSS group exhibit reduced rises slope and diminished integral (per mouse, n = 7,8). **e**, Comparison of spontaneous firing in the DSS group show no significant differences in the additional features (per mouse, n = 14, 10).

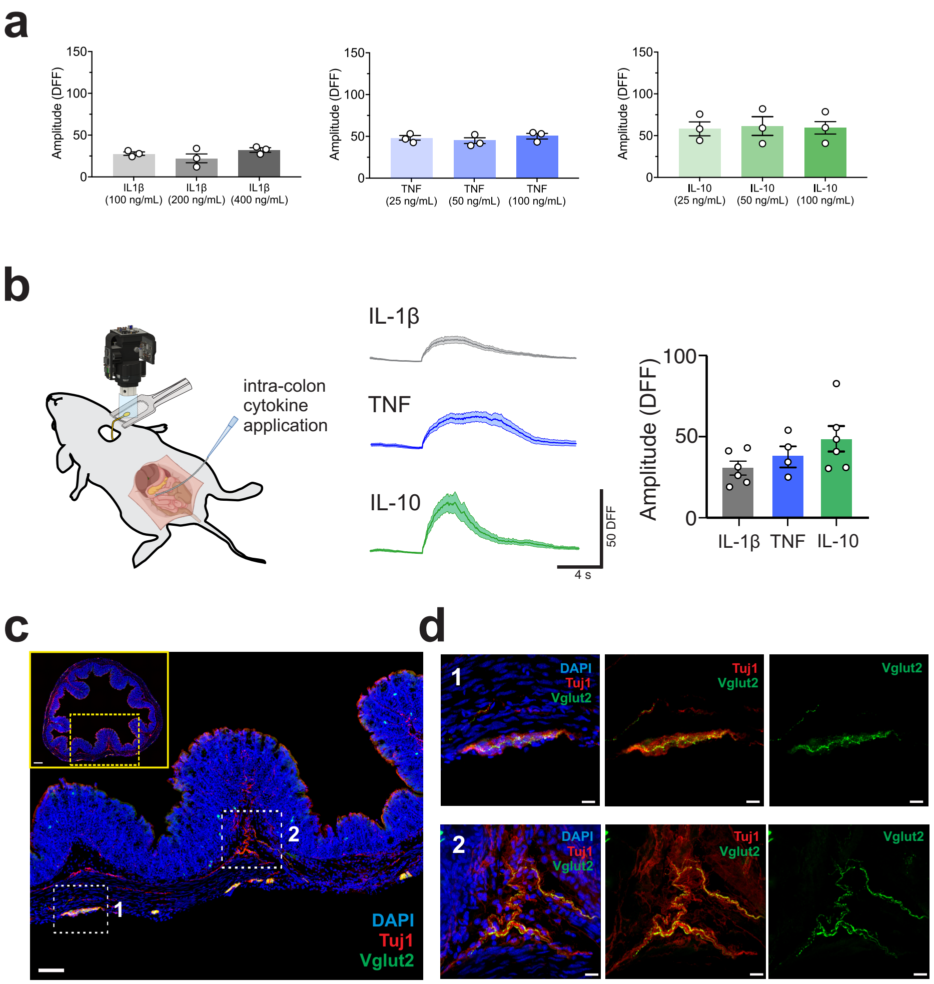

Supplementary Figure 3. **a**, Application of different doses of the same cytokine shows no significant change in the amplitude of cytokine-evoked responses at different concentrations (IL-1β, n = 3 mice per dose, number of responses = 20, 21, 15; TNF, 3 mice per dose, number of responses = 20, 24, 24; IL-10, n = 3 mice per dose, number of responses = 27, 35, 32). **b**, To examine nodose ganglia neuronal responses to cytokines in an end-organ, we applied cytokines within the proximal colon. Left: Schematic shows the experimental setup for these experiments. Center: Cytokine-specific neural responses (mean±SEM) are shown for each of the three cytokines. Right: mean DFF amplitudes are plotted for each cytokine concentration (IL-1, n = 6 mice, number of responses = 49; TNF, n = 4 mice, number of responses = 58; IL-10, n = 6 mice, number of responses = 40). Part of the schematic was created in BioRender. Chang, E. (2025) https://BioRender.com/ayndny9. **c**, Cross-section of the proximal colon from a *Vglut2-GCaMP6f* mouse shows co-localization of glutamatergic Tuj1-positive nerve fibers in the myenteric plexus (Box 1) in addition to the mucosal and submucosal layers of the colon (Box 2). Inset scale bar, 200 µm. Scale bar, 100 µm. **d**, Higher magnification images of the myenteric plexus (Box 1) and the mucosal and submucosal layers (Box 2), showing glutamatergic nerve fibers. Scale bars, 20 µm. Miniscope schematic image in **b** from the UCLA Miniscope project.

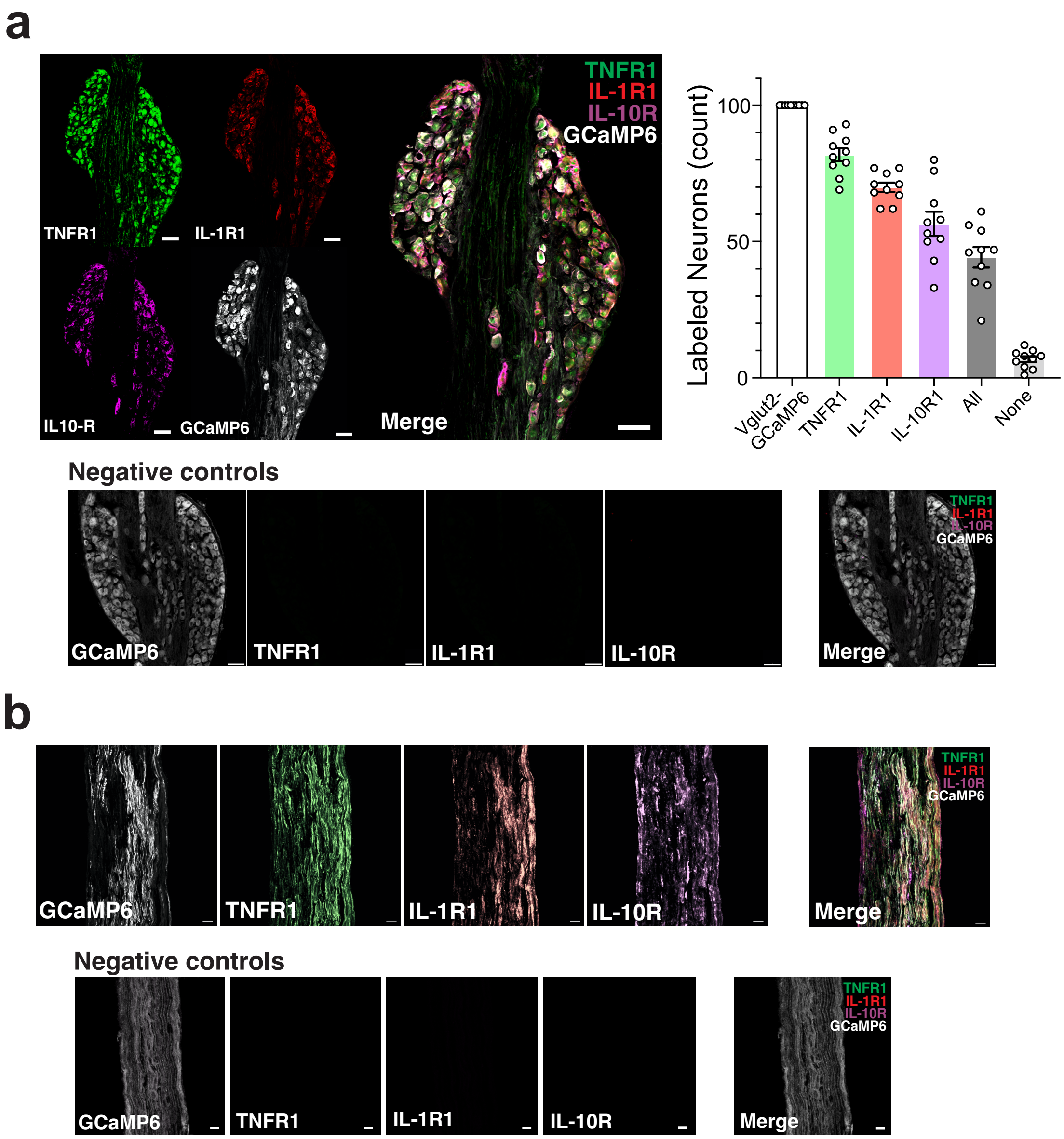

Supplementary Figure 4. Multiplexed labeling of cytokine receptors on the nodose ganglia and vagus nerve. a, Multiplex immunohistochemistry shows Il-1R1, TNFR1, and IL-10R labeling on populations of nodose ganglia neurons (per section, n = 10 from 5 mice). Quantification of each receptor type expressed on VGlut2-GCamp6f nodose ganglia neurons was analyzed using Zen Blue. Scale bar, 50 μm. **b**, Representative images of longitudinal vagus nerve sections also show expression of the three cytokine receptors and Vglut2-GCaMP6f. Scale bar, 20 μm.

Supplementary Table 1. Key Resources

REAGENT or RESOURCE	SOURCE	IDENTIFIER
**Antibodies**		
Mouse TNFR1 Monoclonal Antibody	Proteintech	60192-1-Ig
Rabbit TNFR1 Polycloncal Antibody	ThermoFisher	PA5-119489
Rabbit IL1RA Monoclonal Antibody	Abcam	Ab124962
Rat IL-10RA Monoclonal Antibody	Abcam	Ab33738
Rabbit IL10RA Polyclonal Antibody	ThermoFisher	PA5-109852
Rabbit, Recombinant Anti-PHOX2B antibody [EPR14423] - C-terminal	Abcam	Ab183741
Rabbit, Recombinant Alexa Fluor® 647Anti-PHOX2B antibody [EPR14423] - C	Abcam	Ab311130
Rabbit, Abe95, Anti-PRDM12 Antibody	EMD Millipore	ABE95
FlexAble CoraLite Plus 555 Antibody Labeling Kit for Mouse IgG1	Proteintech	KFA022
Anti-Green Fluorescent Protein Antibody	Aves Labs	GFP-1010
Anti-GFP Antibody	GeneTex	GTX26673
Beta-III-Tubulin Antibody (Tuj1)	Abcam	Ab18207
FlexAble CoraLite® Plus 550 Antibody Labeling Kit for Rabbit IgG	Proteintech	KFA002
FlexAble CoraLite® Plus 647 Antibody Labeling Kit for Rabbit IgG	Proteintech	KFA003
Donkey anti-Rabbit IgG (H+L) Highly Cross-Adsorbed Secondary Antibody, Ale	ThermoFisher	A48258
Donkey anti-Rabbit IgG (H+L) Highly Cross-Adsorbed Secondary Antibody, Ale	ThermoFisher	A32795
Donkey anti Rabbit IgG (H+L) Secondary Antibody, Alexa Fluor 568	ThermoFisher	A10042
Donkey anti-Rat IgG (H+L) Highly Cross-Adsorbed Secondary Antibody, Alexa	ThermoFisher	A48272
AffiniPure Fab Fragment Donkey Anti-Mouse IgG (H+L)	Jackson ImmunoResearch	715-007-003
Fluoromount-G® Mounting Medium, Liquid		
Donkey anti-Chicken IgY (H+L) Highly Cross Adsorbed Secondary Antibody, A	ThermoFisher	A78948
Donkey anti-Goat IgG (H+L) Highly Cross-Adsorbed Secondary Antibody, Alex	ThermoFisher	A32814
NeuroTrace 435/455	ThermoFisher	N21479
Rabbit IgG, monoclonal [EPR25A] - Isotype Control	Abcam	
Rat IgG1 Isotype Control	R&D Systems	
Mouse IgG1 Isotype Control	R&D Systems	
Normal Donkey Serum	SouthernBiotech	
Vector TrueView Autofluorescence Quenching Kit	Vector Laboratories	SP-8400
**Biological samples**		
Normal Donkey Serum	SouthernBiotech	0030-01
**Chemicals, peptides, and recombinant proteins**		
Molecular Probes Neurotrace 435/455 Blue Fluorescent Nissl Stain	ThermoFisher	N21479
Dextran Sulfate Sodium Salt (DSS)	MP Biomedicals	216011080
**Critical commercial assays**		
U-plex custom multiplex immunoassay (mouse)	Meso Scale Discovery	K15069M-2
**Deposited data**		
RNA-sequencing data	Singulomics	NCBI GEO GSE294447
**Experimental models: Organisms/strains**		
C57BL/6J	Jackson Laboratory	664
B6J.129S6(FVB)-Slc17a6tm2(cre)Lowl/MwarJ	Jackson Laboratory	28863
B6J.Cg-Gt(ROSA)26Sortm95.1(CAG-GCaMP6f)Hze/MwarJ	Jackson Laboratory	28865
**Software and algorithms**		
Python 3	Python 3.12.3	www.python.org
CaImAn: Python toolbox for large-scale Calicum Imaging Analysis	Flatiron Intitute	https://github.com/flatironinstitute/CaImAn
FFMPEG	FFMPEG team	https://ffmpeg.org/
Custom Post-CaImAn Analysis Pipeline	This study	Github repository
Zeiss Microscopy Software 3.9	Carl Zeiss Microscopy	Zen (blue edition)
Image-Pro Software 10.2	Media Cybernetics	
