# Supplementary material for: Neural representation of cytokines by vagal sensory neurons
Source: Nat Commun. 2025 Apr 24;16:3840. doi: 10.1038/s41467-025-59248-6 (PMC12019601; doi:10.1038/s41467-025-59248-6)
Supplement: Supplementary file 2 — Description of Additional Supplementary Files [file 41467_2025_59248_MOESM2_ESM.pdf]

Description of Additional Supplementary Files

File name: Supplementary Movie 1
Description: Neuronal responses to cytokines. Calcium imaging videos show cytokine-specific neural responses to interleukin (IL-1β), tumor necrosis factor (TNF), and interleukin-10 (IL-10). Representative example video of three separate calcium imaging experiments demonstrating real-time cytokine-specific neuronal responses to IL-1β, TNF, and IL-10 with DFF filter applied. Videos are shown at 7.5x speed. Each video begins with a baseline period prior to cytokine application on the nerve when "ON" appears. Neurons that are active during the ON period, but not baseline, are analyzed as cytokine-specific responses.

File name: Supplementary Movie 2
Description: Baseline activity of control and DSS-colitis nodose ganglia. Calcium imaging videos show representative baseline spontaneous activity of nodose ganglia neurons from control and DSS-colitis mice at peak disease, day 7. Videos are shown at 7.5x speed.
