# Supplementary material for: Neural representation of cytokines by vagal sensory neurons
Source: Nat Commun. 2025 Apr 24;16:3840. doi: 10.1038/s41467-025-59248-6 (PMC12019601; doi:10.1038/s41467-025-59248-6)
Supplement: Supplementary file 5 — Reporting Summary [file 41467_2025_59248_MOESM5_ESM.pdf]

nature portfolio

Corresponding author(s): Eric H Chang, Kevin J Tracey

Last updated by author(s): Mar 31, 2025

Reporting Summary

Nature Portfolio wishes to improve the reproducibility of the work that we publish. This form provides structure for consistency and transparency in reporting. For further information on Nature Portfolio policies, see our Editorial Policies and the Editorial Policy Checklist.

Statistics

For all statistical analyses, confirm that the following items are present in the figure legend, table legend, main text, or Methods section.

n/a	Confirmed	
☐	☒	The exact sample size (*n*) for each experimental group/condition, given as a discrete number and unit of measurement
☐	☒	A statement on whether measurements were taken from distinct samples or whether the same sample was measured repeatedly
☐	☒	The statistical test(s) used AND whether they are one- or two-sided<br>*Only common tests should be described solely by name; describe more complex techniques in the Methods section.*
☒	☐	A description of all covariates tested
☐	☒	A description of any assumptions or corrections, such as tests of normality and adjustment for multiple comparisons
☐	☒	A full description of the statistical parameters including central tendency (e.g. means) or other basic estimates (e.g. regression coefficient) AND variation (e.g. standard deviation) or associated estimates of uncertainty (e.g. confidence intervals)
☐	☒	For null hypothesis testing, the test statistic (e.g. *F*, *t*, *r*) with confidence intervals, effect sizes, degrees of freedom and *P* value noted<br>*Give P values as exact values whenever suitable.*
☒	☐	For Bayesian analysis, information on the choice of priors and Markov chain Monte Carlo settings
☒	☐	For hierarchical and complex designs, identification of the appropriate level for tests and full reporting of outcomes
☒	☐	Estimates of effect sizes (e.g. Cohen's *d*, Pearson's *r*), indicating how they were calculated

Our web collection on statistics for biologists contains articles on many of the points above.

Software and code

Policy information about availability of computer code

Data collection	Immunohistochemistry and histology images were collected using a Zeiss LSM 880 confocal microscope and a Keyence BZX-810 microscope. Calcium imaging data was acquired using a Miniscope (v4) with DAQ recording software.
Data analysis	Zeiss Zen microscopy software and FIJI/ImageJ plugin were used to quantify and analyze histology images. A modified version of the Calcium Imaging Analysis pipeline (Giovannuci et al, 2019) was used to analyze fluorescence imaging data, additional details and relevant code available in Huerta et al., 2023. GraphPad Prism 10.2.1 was used for statistical analysis and plotting.

For manuscripts utilizing custom algorithms or software that are central to the research but not yet described in published literature, software must be made available to editors and reviewers. We strongly encourage code deposition in a community repository (e.g. GitHub). See the Nature Portfolio guidelines for submitting code & software for further information.

Data

Policy information about availability of data

All manuscripts must include a data availability statement. This statement should provide the following information, where applicable:

- Accession codes, unique identifiers, or web links for publicly available datasets
- A description of any restrictions on data availability
- For clinical datasets or third party data, please ensure that the statement adheres to our policy

All data supporting the findings described in this manuscript are available in the article and its Supplementary Information files. Source data are provided with the paper. Other data that support the findings are available from the corresponding authors upon request.

Research involving human participants, their data, or biological material

Policy information about studies with human participants or human data. See also policy information about sex, gender (identity/presentation), and sexual orientation and race, ethnicity and racism.

Reporting on sex and gender	N/A
Reporting on race, ethnicity, or other socially relevant groupings	N/A
Population characteristics	N/A
Recruitment	N/A
Ethics oversight	N/A

Note that full information on the approval of the study protocol must also be provided in the manuscript.

Field-specific reporting

Please select the one below that is the best fit for your research. If you are not sure, read the appropriate sections before making your selection.

☒ Life sciences ☐ Behavioural & social sciences ☐ Ecological, evolutionary & environmental sciences

For a reference copy of the document with all sections, see nature.com/documents/nr-reporting-summary-flat.pdf

Life sciences study design

All studies must disclose on these points even when the disclosure is negative.

Sample size	Sample sizes were chosen based on prior experience with similar protocols, procedures, and a prior publication using these methods (Huerta et al., 2023).
Data exclusions	Outliers in the data sets were identified using statistical software (GraphPad Prism 10.2.1). Other reasons for data exclusion included any technical errors or if any image samples were out-of-focus or had inappropriate acquisition settings.
Replication	To ensure the reproducibility of the experimental results, we included detailed methods, sources of reagents, and protocols for all of the described experiments. Individual data points and error bars are presented in the figures and sample sizes are provided for the groups. All experiments were carried out in at least duplicate on separate days and experimental controls were performed on each day. All findings were replicated before being included in the final dataset.
Randomization	All mice were randomly assigned to different groups.
Blinding	Experimenter blinding was used whenever possible. For the calcium imaging experiments, experimenters were not blinded to the specific cytokines being applied although the subsequent data analysis was performed by an individual blinded to that information. Quantification of immunohistochemistry images and DSS-colitis tissue histology was performed in a blinded fashion by at least two different individuals.

Reporting for specific materials, systems and methods

We require information from authors about some types of materials, experimental systems and methods used in many studies. Here, indicate whether each material, system or method listed is relevant to your study. If you are not sure if a list item applies to your research, read the appropriate section before selecting a response.

Materials & experimental systems

n/a	Involved in the study
	x Antibodies
x	Eukaryotic cell lines
x	Palaeontology and archaeology
	x Animals and other organisms
x	Clinical data
x	Dual use research of concern
x	Plants

Methods

n/a	Involved in the study
x	ChIP-seq
x	Flow cytometry
x	MRI-based neuroimaging

Antibodies

Antibodies used

Mouse TNFR1 Monoclonal Antibody, Proteintech, 60192-1-Ig
Rabbit IL1RA Monoclonal Antibody, Abcam Ab124962
Rat IL-10RA Monoclonal Antibody, Abcam Ab33738
Rabbit, Recombinant Anti-PHOX2B antibody [EPR14423] - C-terminal, Abcam Ab183741
Rabbit, Recombinant Alexa Fluor® 647Anti-PHOX2B antibody [EPR14423] - C-terminal, Abcam Ab311130
Rabbit, Abe95, Anti-PRDM12 Antibody, EMD Millipore ABE95
FlexAble CoraLite Plus 555 Antibody Labeling Kit for Mouse IgG1, Proteintech KFA022
FlexAble CoraLite® Plus 550 Antibody Labeling Kit for Rabbit IgG, Proteintech KFA002
Donkey anti-Rabbit IgG (H+L) Highly Cross-Adsorbed Secondary Antibody, Alexa Fluor™ Plus 405, ThermoFisher A48258
Donkey anti-Rabbit IgG (H+L) Highly Cross-Adsorbed Secondary Antibody, Alexa Fluor™ Plus 647, ThermoFisher A32795
Donkey anti Rabbit IgG (H+L) Secondary Antibody, Alexa Fluor 568, ThermoFisher A10042
Donkey anti-Rat IgG (H+L) Highly Cross-Adsorbed Secondary Antibody, Alexa Fluor™ Plus 647, ThermoFisher A48272
AffiniPure Fab Fragment Donkey Anti-Mouse IgG (H+L), Jackson ImmunoResearch 715-007-003

Validation

All the primary and secondary antibodies used in this study were validated with positive and negative controls within this study (when possible), by prior studies, and by the antibody manufacturers. Whenever possible, we used knockout-validated antibodies. Links to the manufacturer data and specifications sheet for each antibody is provided below:

https://www.ptglab.com/products/TNFR1-Antibody-60192-1-Ig.htm
https://www.abcam.com/en-us/products/primary-antibodies/il-1ra-antibody-epr6483-ab124962
https://www.abcam.com/en-an/products/primary-antibodies/il-10ra-antibody-1b13a-ab33738
https://www.abcam.com/en-us/products/primary-antibodies/phox2b-antibody-epr14423-c-terminal-ab183741
https://www.abcam.com/en-us/products/primary-antibodies/alexa-fluor-647-phox2b-antibody-epr14423-c-terminal-ab311130
https://www.emdmillipore.com/US/en/product/Anti-PRDM12-Antibody,MM_NF-ABE95
https://www.ptglab.com/products/FlexAble-CoraLite-Plus-555-Antibody-Labeling-Kit-for-Mouse-IgG1-KFA022.htmhttps://www.ptglab.com/products/FlexAble-CoraLite-Plus-555-Antibody-Labeling-Kit-for-Rabbit-IgG-KFA002.htm
https://www.thermofisher.com/antibody/product/Donkey-anti-Rabbit-IgG-H-L-Highly-Cross-Adsorbed-Secondary-Antibody-Polyclonal/A48258
https://www.thermofisher.com/antibody/product/Donkey-anti-Rabbit-IgG-H-L-Highly-Cross-Adsorbed-Secondary-Antibody-Polyclonal/A32795
https://www.thermofisher.com/antibody/product/Donkey-anti-Rabbit-IgG-H-L-Highly-Cross-Adsorbed-Secondary-Antibody-Polyclonal/A10042
https://www.thermofisher.com/antibody/product/Donkey-anti-Rat-IgG-H-L-Highly-Cross-Adsorbed-Secondary-Antibody-Polyclonal/A48272
https://www.jacksonimmuno.com/catalog/products/715-007-003

Animals and other research organisms

Policy information about studies involving animals; ARRIVE guidelines recommended for reporting animal research, and Sex and Gender in Research

Laboratory animals

Experiments were carried out using adult mice (male and female) between 2 to 8 months of age, with the exception of DSS-colitis experiments which only used male mice. Mice were housed on a 12:12 hour reverse light/dark cycle at 22o C and relative humidity of 30-70%. Water was available ad libitum. We created VGLUT2–GCaMP6f mice by crossing homozygous VGLUT2-ires-Cre (Jax#028863) mice with homozygous Ai95D, also known as ROSA-GCaMP6f (Jax#028865) mice.

Wild animals

This study did not involve wild animals.

Reporting on sex

The findings apply to both male and female mice, with the exception of the DSS-colitis data which was collected only from male mice.

Field-collected samples

This study did not involve any field-collected samples.

Ethics oversight

All animals experiments were performed in accordance with the National Institutes of Health (NIH) guidelines for the Care and Use of

Laboratory animals under protocols approved by the Institutional Care and Use Committee (IACUC) of the The Feinstein Institutes for Medical Research.

Note that full information on the approval of the study protocol must also be provided in the manuscript.

Plants

Seed stocks	N/A
Novel plant genotypes	N/A
Authentication	N/A
